# Understanding how perceptions of tobacco constituents and the FDA relate to effective and credible tobacco risk messaging: A national phone survey of U.S. adults, 2014–2015

**DOI:** 10.1186/s12889-016-3151-5

**Published:** 2016-06-23

**Authors:** Marcella H. Boynton, Robert P. Agans, J. Michael Bowling, Noel T. Brewer, Erin L. Sutfin, Adam O. Goldstein, Seth M. Noar, Kurt M. Ribisl

**Affiliations:** Department of Health Behavior, Gillings School of Global Public Health, University of North Carolina at Chapel Hill (UNC), CB #7440, Chapel Hill, NC 27599-7440 USA; Lineberger Comprehensive Cancer Center, UNC, CB #7295, Chapel Hill, NC 27599-7295 USA; Carolina Survey Research Laboratory, UNC, 730 Martin Luther King Jr Blvd, Chapel Hill, NC 27514 USA; Department of Biostatistics, Gillings School of Global Public Health, CB #7420, 3101 McGavran-Greenberg Hall, Chapel Hill, NC 27599-7420 USA; Department of Family Medicine, School of Medicine, UNC, CB #7595, Manning Drive, Chapel Hill, NC 27599-7595 USA; Department of Social Sciences and Health Policy, Wake Forest School of Medicine, Medical Center Boulevard, Winston-Salem, NC 27157 USA; School of Media and Journalism, UNC, CB #3365, Chapel Hill, NC 27599-7440 USA

**Keywords:** Tobacco use, Constituents, Cigarette smoking, Non-cigarette tobacco product, Communication

## Abstract

**Background:**

The passage of the 2009 Family Smoking Prevention and Tobacco Control Act has necessitated the execution of timely, innovative, and policy-relevant tobacco control research to inform Food and Drug Administration (FDA) regulatory and messaging efforts. With recent dramatic changes to tobacco product availability and patterns of use, nationally representative data on tobacco-related perceptions and behaviors are vital, especially for vulnerable populations.

**Methods:**

The UNC Center for Regulatory Research on Tobacco Communication conducted a telephone survey with a national sample of adults ages 18 and older living in the United States (U.S.). The survey assessed regulatory relevant factors such as tobacco product use, tobacco constituent perceptions, and tobacco regulatory agency credibility. The study oversampled high smoking/low income areas as well as cell phone numbers to ensure adequate representation among smokers and young adults, respectively. Coverage extended to approximately 98 % of U.S. households.

**Results:**

The final dataset (*N* = 5,014) generated weighted estimates that were largely comparable to other national demographic and tobacco use estimates. Results revealed that over one quarter of U.S. adults, and over one third of smokers, reported having looked for information about tobacco constituents in cigarette smoke; however, the vast majority was unaware of what constituents might actually be present. Although only a minority of people reported trust in the federal government, two thirds felt that the FDA can effectively regulate tobacco products.

**Conclusions:**

As the FDA continues their regulatory and messaging activities, they should expand both the breadth and availability of constituent-related information, targeting these efforts to reach all segments of the U.S. population, especially those disproportionately vulnerable to tobacco product use and its associated negative health outcomes.

## Background

Tobacco use is the leading cause of preventable death and disease in the United States (U.S.). Morbidity from smoking-related causes is estimated at more than 480,000 deaths per year, which account for 1 out of 5 deaths in the U.S. [1] Cigarettes, the most commonly used tobacco product by adults, have been causally linked with numerous negative health outcomes, including multiple types of cancer, cardiovascular disease, respiratory ailments, and infection [2]. Although the underlying causes are not wholly clear, members of certain stigmatized and vulnerable groups in the U.S., such as those living in poverty and sexual minorities, are disproportionately affected by these negative tobacco-related consequences [3–5]. One of the major reasons cigarettes are harmful to health is the presence of myriad harmful and potentially harmful constituents in cigarette smoke, many of which are known toxicants or carcinogens [6].

Due to local, state, and national education and policy efforts, cigarette smoking has precipitously decreased from over 42 % of the adult population in 1965–17 % in 2014 [7, 8]. In recent years, declines in cigarette smoking have been somewhat offset by increases in use of non-cigarette tobacco products (NCTPs), with the greatest uptake primarily observed for adolescents and young adults [9, 10]. Some NCTPs, such as cigars, have long been available to the public and are a known health hazard. [11] Other products, such as electronic vaping devices, are relatively novel, and as a result have unknown consequences for public health [12]. A growing body of evidence is finding that, like cigarettes, many NCTPs contain harmful and potentially harmful constituents [11, 13, 14]. Considering the substantial health harms of cigarette and NCTP use, more research is needed to inform effective tobacco regulatory and communication efforts.

### Tobacco policy and communication

In 2009, the landmark passage of the Family Smoking Prevention and Tobacco Control Act (FSPTCA) granted the Food and Drug Administration (FDA) the power to regulate tobacco products (Public Law 111–31). Since the passage of the FSPTCA, the FDA has enacted and enforced multiple regulations related to the marketing, manufacturing, and distribution of cigarettes, certain cigarette-related products, and smokeless tobacco. [15] On May 5, 2016 the FDA expanded their regulatory authority to include additional tobacco products, including electronic cigarettes, hookah, and cigars [16, 17]. As part of their tobacco control efforts, the FDA has implemented education campaigns intended to increase the public’s awareness of the potential health harms of tobacco product uptake and use [18].

Many of the tobacco regulatory and education activities performed by the FDA include messaging and communication elements. For example, Sections 904(d) and (e) of the FSPTCA requires the FDA to publish a list of harmful and potentially harmful constituents for each tobacco product, by quantity within each brand and subbrand, in a format that is both understandable and not misleading [19]. Prior research using an online convenience sample of U.S. adults found that although a few tobacco product constituents were familiar to the public (e.g., nicotine, carbon monoxide), the majority of constituents, such as acrolein and tobacco-specific nitrosamines, were generally unknown [20]. Recent qualitative research has not only replicated the finding that the public is largely unaware of the presence of the majority of tobacco constituents in tobacco product smoke or aerosol, but when presented with such information people would often infer meaning and potential harms by relating constituent names to similar-sounding words (e.g., acetaldehyde sounds similar to acetaminophen) [21, 22]. Given that so many constituent names are foreign to the average person, additional research exploring the public’s awareness and interest in tobacco constituents is needed to inform whether and how the FDA might best communicate constituent-related information [23].

With the FDA now serving in a pivotal role of communicating the potential harms of tobacco product use, it is essential to understand how both tobacco product users and non-users perceive the credibility of the FDA and U.S. government. Given the tobacco industry’s documented targeting of adolescents and other vulnerable groups with potent marketing campaigns [24–26], it is also incumbent upon the FDA to develop and implement messaging optimized to effectively communicate the risks of tobacco use to these populations. Notably, a number of groups most affected by tobacco use and its associated health outcomes have also historically experienced mistreatment by government organizations; examples include individuals with lower levels of education and health literacy, those living in poverty, and sexual minorities [27–30]. Effective risk messaging and product labeling from credible information sources will help ensure that the public, especially vulnerable populations, adequately understand the risks of both tobacco product use and the presence of harmful and potentially harmful constituents.

### The current study

In order to build a base of policy relevant tobacco-related research, the FDA, in partnership with the National Institutes of Health, recently funded 14 Tobacco Centers of Regulatory Science (TCORS). As part of this large research effort, our TCORS Center for Regulatory Research on Tobacco Communication (CRRTC) conducted a nationally-representative phone survey of U.S. adults. The current paper reports the methods and sample characteristics from this national phone survey. We compare our demographic and tobacco use estimates to other validated national estimates in order to assess whether our weighted sample is nationally representative. Additionally, we examine the responses for several of the constituent and credibility-related items, discussing the implications of the overall estimates as well as differences observed for certain key groups. Taken together the findings lay a foundation for future empirical work that directly informs how perceptions of tobacco constituents and the FDA relate to effective and credible tobacco risk messaging.

## Method

### Survey measures

#### Development

Using an iterative survey question generation and revision procedure coordinated among three semi-independent projects, the team developed an instrument assessing tobacco-related product use and perceptions, demographic characteristics, general health, and government organization-related credibility and messaging perceptions. Cognitive interviewing was used at various stages of the measures development process to assess the clarity and construct validity of all new measures.

#### Translation

Because English and Spanish are the two most commonly spoken languages in the U.S. [31], the team developed and administered survey measures in both languages. A dual language translation and validation approach was employed using double measures translation with harmonization and validation. Specifically, two professional bilingual translators of differing national origins each independently translated the English language measures. A third fluent Spanish speaker, who served as the translation coordinator and primary measures reviewer, then met with the two translators; through discussion a final version of the Spanish language measures was produced based on the two independent translations. This measures harmonization approach ensured that the Spanish language word usage and syntax was equally accessible to individuals of all Latino and Spanish backgrounds.

#### Testing

After the wording and order of the survey questions was finalized, a pilot test of the proposed survey instrument was implemented between August 5 and August 18, 2014. Several independent and non-overlapping convenience samples were used in the pilot (*N* = 151). To oversample smokers, half of the samples targeted low income households earning less than $25,000 per year. To boost the number of participants in the pilot who identified as gay, lesbian, or bisexual (GLB), a convenience sample of eleven individuals identifying as GLB were recruited and called as a special batch to test the programming specific to GLB participants. Oversampling of young adults (18–25 years of age) occurred within the household through the application of Poisson sampling techniques where they held higher probabilities of selection. The results of the pilot test were used to inform minor survey item revisions and confirm the accuracy of the survey programming.

#### Tobacco use measures

To maximize the fidelity of the tobacco use measures in this study to other national surveys of tobacco product use, many of the tobacco product-related items were taken directly from the Behavioral Risk Factor Surveillance System (BRFSS) questionnaire [32] or from the Population Assessment of Tobacco and Health (PATH) Study [33]. Individuals were classified as current cigarette smokers if they reported having previously smoked at least 100 cigarettes (i.e., five packs) in their lifetime and were currently smoking some days or every day. Smokers were asked about their past 30 day smoking frequency (number of days), menthol cigarette use (none, some, or all cigarettes), cigarette type (e.g., regular, light, ultralight), typical brand (if any), and quit intentions (0 = *not planning to quit* to 3 = *within the next month*).

Non-cigarette tobacco product (NCTP) use was also assessed, with descriptions of the various NCTPs provided to respondents. If individuals indicated ever use of a particular NCTP, they were subsequently queried on their frequency of use in the past 30 days. For the current analyses NCTP use is defined as past 30 day use of any of the following: electronic cigarettes or vaping devices, little cigars or cigarillos, hookah, chewing tobacco, snus, premium cigars, or any other tobacco product. Any tobacco use was defined as past 30 day NCTP use or an individual reporting cigarette smoking some days or every day.

#### Tobacco constituent measures

A variety of questions related to tobacco constituent information, knowledge, and perceptions were administered. To determine the frequency of information-seeking related to cigarette constituents, participants were asked “Have you ever looked for information on chemicals in cigarettes and cigarette smoke?” As a follow-up question participants were asked “In which 1 of these 3 places would you most like to see information on chemicals in cigarettes and cigarette smoke: on cigarette packs, in stores, or online?” Twenty-four cigarette smoke constituents were selected for assessment by participants. In order to minimize participant burden, the constituents were divided into 6 panels of 4 constituents each, with each participant answering questions for one panel (see Appendix A for the list of constituents by panel).

#### FDA credibility

Multiple items related to FDA credibility were also administered. Participants were asked whether they had ever heard of the FDA and whether they felt the FDA could “effectively regulate tobacco products.” Because sampling efforts were particularly targeted to groups who have historically have been marginalized or exploited by certain U.S. governmental and other authoritative bodies, we also assessed general trust in the government using the item: “How much trust do you have in the federal government?” Responses ranged from 0 = *none at all* to 4 = *a great deal*.

#### Demographics

Characteristics such as gender, age, ethnicity, education, income were assessed primarily using measures from the 2013 BRFSS survey [31] or the 2010 U.S. Census [34]. Race was assessed using the item, “Which one of these groups would you say best represents your race: White, Black or African American, American Indian or Alaska Native, Asian, or Pacific Islander?” Individuals strongly identifying as an unlisted or mixed race were coded as “Other.” Education was assessed using an ordinal scale ranging from 0 = *no schooling completed* to 15 = *doctorate degree*. Numeracy was assessed using a single item adapted from a standard numeracy scale [35]: “In general, which of these numbers shows the biggest risk of getting a disease: 1 in 100, 1 in 1000, or 1 in 10?” Poverty level was determined using the household size and income reported by the respondents and applying the federal poverty numbers available from the U.S. Department of Health and Human Services in 2014. The sexual orientation measure was developed using guidelines provided by the Williams Institute [36], which asked “Do you consider yourself to be (A) straight or heterosexual, (B) gay or lesbian, or (C) bisexual?”

### Sampling and recruitment

Two independent and non-overlapping random digit dialing frames were used in this study with approximately 98 % coverage of all U.S. adult households [37]. To oversample smokers, both frames were stratified by household income and smoking rates at the county-level, where the poorest counties with the highest smoking rates were oversampled. Concordant with prior national tobacco survey studies [38], we oversampled cell phones numbers to maximize counts of young adults. To be considered eligible, a telephone number needed to reach a household with an English- or Spanish-speaking resident 18 years of age or older. Within the landline frame, if more than one eligible adult resided in the household, young adults and smokers were sampled at a higher rate than older adult nonsmokers.

The national survey was conducted between September 15, 2014 and May 31, 2015 and had an average completion time of 25 min. Calls were made Saturday through Thursday between 9 am and 9 pm (local time). Blaise CATI software [39] was used to both manage the sample and collect the data. No numbers were removed from calling until a minimum of 6 (cell phone) to 8 (landline) unsuccessful call attempts were made with at least one weekend, evening, and daytime call attempt. The sample resulted in 5,014 interviews and a weighted response rate (calculated using AAPOR Response Rate 4) of 42 %, a rate which is comparable to the 2012–2013 National Adult Tobacco Survey (44.9 %) [40] and the 2012 BRFSS (45.3 %) [41]. The remaining sample consisted of ineligible numbers (64,410), refusals from eligible households (2,623), or indeterminable eligibility status (41,877). All interviewers completed general and project-specific training before conducting the surveys and were monitored twice fortnightly. Informed consent for participation in the study was obtained verbally from respondents at the time of enrollment. The IRB at the University of North Carolina approved all study procedures and respondents were protected by a certificate of confidentiality.

### Sampling weights and adjustments

A standard three-step sample weighting procedure was followed to produce sampling weights [42]. The base weights were computed using the sampling rate for telephone numbers in each stratum, adjusting for the number of eligible respondents and landline telephone numbers in the household as well as any oversampling of young adults and/or smokers that might have occurred in the landline sample (Step 1). The base weights were then adjusted for differential household-level nonresponse among sampling strata using the inverse of the stratum-specific household-level response rate as the adjustment factor (Step 2). The nonresponse-adjusted household sample weight was then calibrated to population counts as estimated from the American Community Survey [34] sample by implementing the SAS rake and trimming macro [43] on the following variables: census region, age (18–24, 25–44, 45–64, or ≥ 65), education (≤ high school, some college, or bachelor’s degree and higher), gender, ethnicity (Hispanic or non-Hispanic), phone-type (cell or landline) and regional smoking rates. Final weights were normalized to the total sample size [44].

### Analysis

All analyses were conducted using SAS version 9.3 and took the sample design features into account. Weighted sample means and proportions with 95 % confidence intervals incorporating both sampling weight and strata variables were computed using the PROC SURVEYMEANS and PROC SURVEYFREQ procedures. Stratum-specific weighted analyses by subgroup (e.g., smokers, young adults) employed the BY command for the PROC SURVEYMEANS procedure and the TABLE command for the PROC SURVEYFREQ procedure. Weighted analyses of intra-group differences for categorical variables (i.e., comparisons between smokers vs. non-smokers, young adults vs. older adults, etc.) employed *χ*^2^ tests using the CHISQ command. For the continuously scaled trust in the federal government variable, means were generated using PROC SURVEYMEANS and intra-group comparisons were made using PROC SURVEYREG.

## Results

### Demographics

Examination of the weighted estimates revealed a weighted proportion of 50.8 % females and an age range of 18 to 95 years (*M* = 45.9, *SD* = 17.3). The two largest racial groups in this sample were White (68.3 %) and Black/African American (18.3 %). Approximately 14 % of the sample identified as Latino or Hispanic. Young adults (i.e., individuals aged 18–24) comprised 12.7 % of the sample and, based on reported household size and annual income, 14.3 % of the sample was identified as living below the U.S. federal poverty line. In addition, 3.2 % of the sample identified as GLB.

In order to assess the quality of our sampling design we compared this study’s weighted demographic estimates to comparable national point estimates, thereby providing a sense of the relative “representativeness” of our weighted sample. As can been seen in Table 1, across a wide range of demographic factors the majority of estimates from other national surveys or the U.S. Census fall within the 95 % confidence intervals (CI) of the sample’s weighted point estimates. The only noteworthy exceptions were race and ethnicity, which slightly overestimates the proportions of Whites and African Americans and slightly underestimates the proportions of Asians and Latinos. In each case, the difference between the national estimate and the relevant confidence interval bound was no more than 3.5 percentage points.

**Table 1:** Demographic characteristics as compared to U.S. Census and other national surveys, CRRTC national adult (≥18 years) phone survey 2014-2015

	Unweighted	Weighted		National estimate
	% (*n*)	%	95 % CI	%
Gender				
Male	47.3 % (2372)	48.5 %	(46.0-51.0)	49.2 % [34]
Female	52.7 % (2640)	51.5 %	(49.0-54.0)	50.8 % [34]
Age, years	45.9 ± 17.3	46.7	(45.8-47.7)	
Age category				
18-24	14.2 % (711)	12.7 %	(11.2-14.1)	13.1 % [34]
25-44	32.3 % (1612)	33.2 %	(30.8-35.5)	35.0 % [34]
45-64	37.7 % (1883)	36.7 %	(34.3-39.1)	34.7 % [34]
65+	15.8 % (789)	17.5 %	(15.3-19.6)	17.2 [34]
Race				
White	69.7 % (3473)	68.3 %	(65.9-70.6)	62.6 % [34]
Black or African American	19.6 % (978)	18.3 %	(16.3-20.3)	13.2 % [34]
American Indian or Alaska Native	2.7 % (135)	1.9 %	(1.3-2.6)	1.2 % [34]
Asian	2.1 % (104)	2.4 %	(1.8-3.1)	5.3 % [34]
Pacific Islander	0.4 % (21)	0.8 %	(0.3-1.3)	0.2 % [34]
Other or Unknown	5.4 % (270)	8.2 %	(6.8-9.7)	
Ethnicity				
Latino/Hispanic	8.6 % (432)	14.2 %	(12.4-16.0)	17.1 % [34]
Non-Latino/Hispanic	91.4 % (4568)	85.8 %	(84.0-87.6)	82.9 % [34]
Education				
< High school (HS)	10.5 % (524)	11.2 %	(9.2-13.2)	12.3 % [34]
G12 or GED, HS diploma	24.7 % (1232)	31.4 %	(28.8-34.0)	29.6 % [34]
Some college	20.7 % (1034)	20.7 %	(18.8-22.6)	19.4 % [34]
Associate’s degree	9.9 % (496)	10.5 %	(9.0-12.0)	9.4 % [34]
Bachelor’s degree	21.2 % (1060)	15.7 %	(14.3-17.1)	18.9 % [34]
Graduate or professional degree	13.0 % (651)	10.5 %	(9.4-11.6)	10.4 % [34]
Numeracy				
Incorrect or “do not know” numeracy response	32.0 % (1599)	31.9 %	(29.5-34.3)	
Correct numeracy response	68.0 % (3401)	68.1 %	(65.7-70.5)	75.3 % [53]
Household Poverty				
At or above federal poverty level	84.0 % (3901)	85.7 %	(83.8-87.5)	84.6 % [34]
Below federal poverty level	16.0 % (745)	14.3 %	(12.5-16.2)	15.4 % [34]
Sexual Orientation				
Straight or heterosexual	94.3 % (4730)	94.2 %	(93.1-95.3)	
Gay, lesbian, or bisexual	3.8 % (192)	3.2 %	(2.5-3.8)	3.5 % [54]
Other or refused	1.8 % (92)	2.6 %	(1.7-3.5)	
Census Region				
Northeast	10.7 % (537)	18.2 %	(16.3-20.2)	17.9 % [34]
Midwest	19.4 % (972)	21.5 %	(19.3-23.8)	21.7 % [34]
South	53.6 % (2685)	37.1 %	(34.7-39.5)	37.1 % [34]
West	16.3 % (819)	23.1 %	(21.1-25.1)	23.3 % [34]
Tobacco Product Use				
Any tobacco product use, past 30 days	32.6 % (1633)	28.4 %	(26.2-30.6)	25.2 % [40]
No tobacco product use, past 30 days	67.4 % (3381)	71.6 %	(69.4-73.8)	
Current cigarette smoking				
Current smoker	23.0 % (1151)	17.8 %	(16.0-19.6)	18.0 % [40]
Non-smoker	77.0 % (3856)	82.2 %	(80.4-84.0)	
Used ≥ 1 NCTP in past 30 days	20.4 % (1022)	18.6 %	(16.7-20.5)	

[34] US Census 2013–2014 [53]; Galesic & Garcia-Retamero (2010) [54]; Gallup 2013 LGBT poll [40]; CDC’s National Adult Tobacco Survey, Tobacco Product Use Among Adults — United States, 2012–2013

### Tobacco product use

As a result of the oversampling strategy employed in this study, smokers represented 23.0 % (*N* = 1151) of the unweighted sample; however, at 17.8 % the weighted smoking prevalence for the entire sample was effectively identical to the national prevalence estimate (see Table 1). The national estimate for any tobacco product use (25.2 %), which encompassed both cigarette and NCTP use, was within one percentage point of the lower bound of the 95 % CI for our estimate of 28.4 % [40]. Table 2 presents the weighted proportion of smokers for key demographic characteristics. Most of the estimates for our sample fell within the CIs of the U.S. Census or other national estimates, with the remainder falling within 2 percentage points of either the upper or lower confidence bound.

**Table 2:** Percentage of smokers by selected demographic characteristics, CRRTC national adult (≥18 years) phone survey 2014-2015

	Weighted		National estimate	
	%	95 % CI	%	95 % CI
Gender				
Male	18.6 %	(16.1-21.1)	18.8 %	(18.0-19.7)
Female	17.0 %	(14.4-19.7)	14.8 %	(14.0-15.7)
Age category				
18-24	15.4 %	(11.6-22.4)	16.7 %	(14.0-19.3)
25-44	22.3 %	(18.7-26.0)	20.0 %	(19.1-21.0)
45-64	19.5 %	(16.4-22.5)	18.0 %	(17.0-19.1)
65+	7.8 %	(5.0-10.5)	8.5 %	(7.7-9.3)
Race				
White	17.6 %	(15.4-19.8)	18.2 %	(18.6-20.2)
Black or African American	21.6 %	(16.7-26.4)	17.5 %	(16.1-18.8)
American Indian or Alaska Native	26.7 %	(12.9-40.4)	29.2 %	(19.7-38.7)
Asian	6.5 %	(2.1-10.9)	9.5 %	(7.7-11.2)
Pacific Islander				
Other or Unknown	16.6 %	(10.7-22.4)	26.8 %	(21.9-31.8)
Ethnicity				
Latino/Hispanic	18.7 %	(16.7-20.7)	11.2 %	(11.0-13.2)
Non-Latino/Hispanic	12.7 %	(8.7-16.7)		
Education				
< High school (HS)	25.8 %	(18.7-32.8)	22.9 %	(21.3-24.5)
G12 or GED, HS diploma	21.9 %	(18.2-25.6)	21.7 %	(20.3-23.0)
Some college	22.3 %	(17.6-27.1)	19.7 %	(18.3-21.1)
Associate’s degree	17.4 %	(12.6-22.2)	17.1 %	(14.5-19.6)
Bachelor’s degree	8.2 %	(5.9-10.4)	7.9 %	(7.1-8.8)
Graduate or professional degree	3.5 %	(1.8-5.2)	5.4 %	(4.5-6.3)
Numeracy				
Incorrect or “do not know” numeracy response	21.4 %	(18.0-24.7)		
Correct numeracy response	16.1 %	(13.9-18.3)		
Household Poverty				
At or above federal poverty level	15.4 %	(13.5-17.3)	15.2 %	(14.6-15.9)
Below federal poverty level	29.3 %	(23.9-34.7)	29.2 %	(27.5-31.0)
Sexual Orientation				
Straight or heterosexual	17.5 %	(15.7-19.4)	17.6 %	(16.9-18.2)
Gay, lesbian, or bisexual	24.4 %	(16.1-32.6)	26.3 %	(24.6-28.1)
Other or refused	18.8 %	(3.1-34.5)		
Census Region				
Northeast	16.9 %	(12.2-21.7)	15.3 %	(13.9-16.7)
Midwest	20.6 %	(15.8-25.4)	20.7 %	(18.9-22.4)
South	19.2 %	(16.5-21.9)	17.2 %	(16.3-18.1)
West	13.6 %	(10.6-16.6)	13.1 %	(12.1-14.2)
Used ≥ 1 NCTP in past 30 days	44.1 %	(38.8-49.4)		

National estimates taken from Jamal et al., 2014 [8]

Consistent with the literature, smoking rates were notably higher for respondents reporting less education, low literacy, and living below the federal poverty line. Furthermore, cigarette smoking was relatively higher for GLBs, Native Americans, and NCTP users, a finding also concordant with prior research [8]. The only notable differences between our estimates and other national estimates of tobacco use were among Black (vs. White) and Latinos (vs. non-Latinos), which were modestly overestimated.

Table 3 presents cigarette use characteristics for the smokers. The majority (73.5 %) reported smoking every day in the past 30 days. A little over a third of respondents (38.8 %) reported only smoking menthols in the past 30 days, and another 15.8 % smoked some menthols during that time. Regular or full flavor cigarettes were the most commonly smoked type (58.5 %), followed by light or mild, (29.4 %). The Centers for Disease Control and Prevention (CDC) estimates for the top four cigarette brands in the U.S. are 41 % Marlboro, 12 % Newport, 8 % Pall Mall, and 8 % Camel [45]. Our weighted estimates were largely equivalent: Marlboro, 38.2 %, CI [32.7, 43.7], Newport, 20.1 %, CI [15.9, 24.3], Pall Mall, 7.0 %, CI [4.0, 9.9], and Camel, 6.3 %, CI [4.1, 8.4]. Notably, a majority of smokers (81.5 %) reported planning to quit sometime in the future.

**Table 3:** Current smoker cigarette use characteristics, adults ≥ 18 years, CRRTC national adult phone survey 2014-2015

	Weighted estimates	
	%	95 % CI
Number of days smoked in the past 30 days	25.3 [0–30]	(24.5-26.1)
Past 30 day smoking frequency		
0 days	0.8 %	(0.3-1.4)
1 or 2 days	2.2 %	(1.0-3.3)
3 to 5 days	5.8 %	(3.6-7.9)
6 to 9 days	1.9 %	(0.6-3.3)
10 to 19 days	7.9 %	(5.4-10.5)
20 to 29 days	7.9 %	(3.4-12.3)
All 30 days	73.5 %	(68.3-78.7)
Past 30 day menthol use		
All cigarettes smoked were menthols	38.8 %	(33.0-44.6)
Some cigarettes smoked were menthols	15.8 %	(11.9-19.7)
No cigarettes smoked were menthols	45.5 %	(40.0-50.9)
Cigarette Type		
Regular or full flavor	58.5 %	(53.3-63.7)
Light or mild	29.4 %	(24.3-34.5)
Ultra light	7.3 %	(4.7-9.9)
Other or unspecified	4.8 %	(2.4-7.3)
Cigarette Brand		
Marlboro	38.2 %	(32.7-43.7)
Newport	20.1 %	(15.9-24.3)
Pall Mall	7.0 %	(4.0-9.9)
Camel	6.3 %	(4.1-8.4)
L&M	3.2 %	(1.7-4.6)
Maverick	2.6 %	(0.2-5.0)
Pyramid	2.6 %	(0.2-1.6)
American Spirit	1.6 %	(0.7-2.5)
Kool	1.5 %	(0.6-2.5)
Virginia Slims	0.9 %	(1.3-3.3)
Doral	0.8 %	(0.2-1.4)
Salem	0.8 %	(0.1-1.2)
Winston	0.7 %	(1.8-5.5)
Parliament	0.6 %	(0.1-1.5)
Benson & Hedges	0.4 %	(0.0-0.9)
Generic or least expensive	2.3 %	(3.3-7.0)
Roll your own	3.7 %	(0.0-0.9)
Other brand	6.8 %	(4.8-8.9)
Quit Intentions		
Within the next month	23.4 %	(18.8-27.9)
Within the next 6 months	24.8 %	(19.4-30.2)
Sometime in the future beyond 6 months	32.3 %	(27.4-37.2)
Not planning to quit	19.5 %	(15.7-23.3)

**Table 4:** Subset of communication-related variables – CRRTC national adult phone survey 2014-2015

	Weighted proportion or *M* with 95 % confidence interval						
	Total	Smokers	Young adults	Low education	Low numeracy	Living in poverty	GLBs
Information Seeking							
Have you ever looked for information on chemicals in cigarettes and cigarette smoke?							
Yes	27.5 % (25.4-29.7)	**34.3 % (28.8-39.8)**	**37.2 % (31.6-42.8)**	**22.2 % (18.6-25.7)**	26.7 % (22.7-30.6)	25.7 % (19.7-31.6)	30.1 % (21.0-39.1)
No	72.5 % (70.3-74.6)	**65.7 % (60.2-71.2)**	**62.8 % (57.2-68.4)**	**77.8 % (74.3-81.4)**	73.3 % (69.4-77.3)	74.3 % (68.4-80.3)	69.9 % (60.9-79.0)
In which 1 of these 3 places would you most like to see information on chemicals in cigarettes and cigarette smoke?							
On cigarette packs	54.8 % (52.4-57.3)	57.2 % (51.9-62.6)	**46.8 % (40.9-52.6)**	55.4 % (50.9-60.0)	53.9 % (49.5-58.3)	54.3 % (47.4-61.2)	46.9 % (36.4-57.3)
In stores	15.0 % (13.2-16.7)	11.6 % (7.9-15.3)	**14.7 % (10.7-18.7)**	16.8 % (13.6-20.0)	16.0 % (12.9-19.2)	18.3 % (13.2-23.5)	17.2 % (10.3-24.1)
Online	28.7 % (26.5-30.9)	28.8 % (24.2-33.4)	**38.2 % (32.5-43.8)**	26.2 % (22.3-30.0)	28.5 % (24.6-32.5)	25.5 % (19.7-31.4)	35.8 % (26.1-45.4)
Don't know, refused, or doesn’t want information	1.5 % (0.9-2.08)	2.4 % (1.0-3.7)	**0.2 % (0.0-0.8)**	1.6 % (0.5-2.7)	1.5 % (0.56-2.46)	1.8 % (0.3-3.3)	0.1 % (0.0-0.3)
Constituent Awareness							
Aware of 0 of 4 constituents in cigarette smoke	37.5 % (35.0-40.1)	36.7 % (31.6-41.8)	32.7 % (27.3-38.2)	**46.4 % (28.9-37.2)**	**42.9 % (38.4-47.4)**	43.1 % (35.7-50.4)	31.3 (22.0-40.7)
Aware of 1 of 4 constituents in cigarette smoke	35.8 % (33.4-38.2)	41.2 % (35.6-46.9)	36.1 % (30.4-41.7)	**33.1 % (35.4-40.9)**	**34.5 % (30.3-38.7)**	34.7 % (28.2-41.2)	38.9 (28.5-49.3)
Aware of 2 of 4 constituents in cigarette smoke	18.7 % (16.7-20.7)	15.8 % (12.2-19.4)	22.7 % (17.9-27.6)	**14.5 % (19.1-23.6)**	**16.0 % (12.9-19.2)**	13.6 % (9.8-17.5)	18.2 (11.0-25.4)
Aware of 3 of 4 constituents in cigarette smoke	5.6 % (4.6-6.5)	3.4 % (1.9-4.8)	5.9 % (3.4-8.4)	**3.3 % (5.9-8.6)**	**4.8 % (3.3-6.3)**	5.7 % (3.0-8.4)	9.3 (3.0-15.7)
Aware of 4 of 4 constituents in cigarette smoke	2.4 % (1.7-3.1)	3.0 % (0.5-5.4)	2.5 % (0.6-4.5)	**2.8 % (1.5-2.9)**	**1.8 % (0.8-2.9)**	2.9 % (0.8-5.1)	2.3 (0.0-5.0)
Knowledge of and Trust for FDA and U.S. Federal Government							
Have you ever heard of the FDA or Food and Drug Administration?							
Yes	94.6 % (93.4-95.8)	95.4 % (93.0-95.8)	**90.9 % (87.7-94.2)**	**89.7 % (87.0-92.3)**	**91.7 % (89.0-94.3)**	**87.5 % (83.0-92.0)**	91.7 % (85.0-98.3)
No	5.4 % (4.2-6.6)	4.6 % (2.5-6.8)	**9.1 % (5.8-12.3)**	**10.3 % (7.7 %-13.0)**	**8.3 % (5.7-11.0)**	**12.5 % (8.0-17.0)**	8.3 % (1.7-15.0)
Can the FDA effectively regulate tobacco products?							
Yes	65.2 % (62.6-67.8)	66.6 % (61.2-72.0)	**79.3 % (74.7-83.9)**	64.9 % (59.9-69.9)	62.1 % (57.4-66.9)	67.8 % (61.3-74.1)	**76.3 % (67.1-85.4)**
No	34.8 % (32.2-37.4)	33.4 % (26.9-37.4)	**20.7 % (74.7-83.9)**	35.1 % (31.9-37.5)	37.9 % (33.1-42.6)	32.2 % (25.8-38.7)	**23.7 % (14.6-32.9)**
How much trust do you have in the federal government? *M score, 0 = none at all - 4 = a great deal*	2.0 (1.9-2.0)	**1.7 (1.6-1.8)**	2.1 (2.0-2.2)	1.9 (1.8-2.0)	2.0 (1.8-2.1)	**2.2 (2.0-2.4)**	2.1 (1.9-2.4)

Note. Point estimates in bold text were found to be significantly different from their respective comparison group (e.g., smokers were compared to non-smokers, young adults compared to older adults, etc.) using either PROC SURVEYFREQ or PROC SURVEYREG to make the comparisons

### Differences in constituent and FDA-related perceptions by vulnerable groups

#### Constituent information

​Table 4 presents tobacco constituent communication findings. More than a quarter of adults (27.5 %) reported having looked for information on tobacco constituents. Of those, higher proportions of smokers (34.3 %) and young adults (37.2 %) had previously looked for this information, as compared to non-smokers (26.1 %, *p* = .004) and older adults (26.0 %, *p* < .0001), respectively. A smaller proportion of individuals with low education reported having previously looked for information on tobacco constituents (22.2 %) as compared to those with greater educational attainment than a high school diploma (31.5 %, *p* < .0001).

When asked where they would most like to see information on tobacco constituents, over half indicated that they would prefer it on cigarette packs (54.8 %) and another quarter most wanted the information available online (28.7 %). There was no difference between smokers and non-smokers for information location preference; however, as compared to older adults (27.4 %), a higher proportion of young adults preferred that constituent information be available online (38.2 %, *p* = .0003).

Over one third of U.S. adults were not aware that any of the four constituents in their survey panel were present in cigarette smoke (37.5 %), and only 8 % knew that at least three of the constituents in their survey panel are present in cigarette smoke. Constituent awareness was lower for the low educational attainment (*p* < .0001) and low numeracy groups (*p* = .02), with over 75 % of both sub-groups not aware of more than 1 constituent in their survey panel being present in cigarette smoke.

### FDA credibility

The vast majority of U.S. adults (94.6 %) reported having heard of the FDA, although awareness was lower for young adults (90.9 %, *p* = .007), those with low education (89.7 %, *p* < .0001), those with low numeracy (91.7 %, *p* = .0009), and those living in poverty (87.5 %, *p* < .0001). The majority of both smokers (66.6 %) and non-smokers (65.0 %) believed that the FDA can effectively regulate tobacco products. The proportions of people endorsing effective FDA tobacco product regulation were even higher for young adults (79.3 %, *p* < .0001) and GLBs (76.3 %, *p* = .04). Of note, young adults were much more likely to identify as GLB as compared to older adults, *χ*^2^(1) = 21.5, *p* < .0005.

In stark contrast to the relative support of the FDA, less than half of U.S. adults (42.9 %) reported feeling some trust in the federal government (i.e., a rating of 3 = *a fair amount* or 4 = *a great deal*). On average, smokers reported less trust in the federal government (*M* = 1.7) as compared to non-smokers, (*M* = 2.0, *p* < .0001). Additionally, individuals living in poverty had greater trust in the government (*M* = 2.2) as compared to those not living in poverty, (*M* = 2.0, *p* = .004).

## Discussion

The passage of the 2009 FSPTCA promised to usher in a new era in tobacco regulation that has enormous implications for improving public health. The funding of 14 TCORS is an important advancement in the field of tobacco regulatory science, with the national phone survey detailed herein offering relevant and timely data that can inform FDA policy and messaging efforts. The survey had a response rate of 42 %, which is on par with other national tobacco surveys. We found that our weighted tobacco use estimates mirrored CDC estimates and U.S. demographic estimates largely fell within the confidence bounds of our sample’s weighted estimates. These finding indicate that our sample weights appropriately adjusted our estimates to reflect those of the U.S. population. These encouraging findings pave the way for additional analyses of data from this dataset, especially as relevant to perceptions of tobacco product constituents, FDA credibility, and tobacco communication.

Because tobacco product marketing and tobacco-related health outcomes disproportionally impact younger and marginalized communities as well as those with a history of tobacco use, we chose to strategically oversample individuals from these groups. Comparisons between the unweighted and weighted estimates in Table 1 showed that we successfully oversampled smokers and young adults as well as achieved comparable proportions for individuals with low educational attainment and those living in poverty—a noteworthy achievement given that these groups tend to be under-represented in national surveys [46]. By obtaining robustly sized sub-samples, it was possible to generate stable group estimates for key groups on a number of tobacco constituent and FDA credibility-related perceptions.

Examination of the constituent-related measures showed that the majority of the U.S. public would like ready access to tobacco constituent information. In fact, our results reveal that groups one might presume to be the least psychologically motivated to search for tobacco constituent information, young adults and smokers, were *most* likely to say that they had previously looked for this information. Moreover, more than 80 % of U.S. smokers report intending to eventually quit smoking, suggesting that many smokers are in the contemplation stage of behavior change and would therefore benefit from greater access to constituent information [47]. Taken together, these findings indicate that the legislatively mandated publication of tobacco constituent information is of great interest to the public, and if executed well, could improve public health. Our results also showed that different groups may prefer different channels of information. For example, older adults preferred constituent information on cigarette packs whereas young adults equally preferred it on packs and online. Given these results, the FDA may want to consider making constituent information available through multiple channels.

Although nearly one third of U.S. adults have actively sought out information about tobacco constituents, the public appears to still be largely unaware of what constituents are contained in cigarette smoke. In the current study, we asked respondents whether they had heard that each of 4 constituents are in cigarette smoke.. As there were six different panels, we ultimately obtained data on 24 unique constituents, all of which appear on the FDA’s full list of 93 harmful and potentially harmful cigarette smoke constituents [48]. With the exception of nicotine, most people were largely unaware of what constituents are present in cigarette smoke. Over one third of respondents were unaware that even one of their listed constituents were present in cigarette smoke, and another third only reported knowing one of their four as being present in cigarette smoke. Future FDA messaging efforts could benefit from including information about the presence and health implications of tobacco constituents.

The data presented herein indicate that for most U.S. adults the FDA is a known entity that is capable of regulating tobacco. In stark contrast, the majority of people in the U.S. report low levels of trust in the “federal government.” In other words, although FDA is a part of the federal government, individuals may not typically think of it as such. Thus, in certain cases identifying the FDA as the source of a counter tobacco message may help to increase the credibility and impact of the message.

### Limitations and future directions

The current study’s strengths include the recruitment of a large, nationally representative sample, targeted oversampling of key vulnerable groups, and the development of psychometrically valid health, tobacco use, and constituent communication items administered in both English and Spanish. Our study largely focused on constituents for which the FDA has signaled that they are most likely to require tobacco manufacturers to report quantity information [49]. However, with well over 5,000 chemicals in tobacco products [6] and 93 that the FDA has already identified as harmful or potentially harmful [48], future messaging efforts will likely expand to include an array of different constituents. Although our findings are consistent with past research showing low levels of awareness for the presence of the majority of constituents in cigarette smoke, future studies exploring awareness of a wider range of constituents would be informative, especially once the FDA releases constituent information for tobacco products. A second limitation is that the unique associations between GLB status, age, and tobacco-related perceptions are somewhat difficult to disentangle because, as compared to older adults, young adults more likely to identify as GLB. There is a great need for more tobacco control research with those who identify as GLB, especially considering that this population has a substantially higher tobacco use rate as compared to their non-GLB peers [50].

Future analyses of our adult phone survey data will examine several key tobacco communication issues, and portions of the survey will be repeated in two years’ time to examine potential temporal changes. One important area meriting further exploration is how the U.S. public perceives tobacco product use in the context of learning about constituents in cigarettes and NCTPs. In other words, there are a wide range of possible constituents that the FDA could message on; it would be instructive to explore whether certain types of constituents have a greater or lesser impact on tobacco use risk perceptions. Relatedly, given the highly technical names of many onstituent names, it would be of value to examine what contextual information might be important to include with constituent disclosures to make clear the risks associated with their presence in cigarettes and NCTPs (e.g., what health effects are caused by particular constituents).

Another critical tobacco communication issue is the public’s perceptions of tobacco messaging agencies and their public health campaigns [51], especially as related to perceived source credibility [52]. Our preliminary findings indicate that different vulnerable groups have varied perceptions of the sources of tobacco health messages (e.g., FDA, CDC), suggesting that the source of messages may need to be emphasized in different ways, depending on the target audience. Future research that delves into the cognitive mechanisms underlying these group differences in government organization credibility would be informative.

## Conclusions

As the FDA moves forward with its tobacco policy and communication efforts, the positive impact on tobacco perceptions and use can be maximized by incorporating empirical evidence considering issues such as constituent perceptions and tobacco regulatory agency messaging credibility. Additional national survey work in the U.S. context is needed in order to monitor the public’s response to FDA communications as well as to identify changing patterns of tobacco-related perceptions and use, especially for vulnerable populations.

## Abbreviations

BRFSS, behavioral risk factor surveillance system; CDC, centers for disease control and prevention; CI, 95 % confidence interval; CRRTC, center for regulatory research on tobacco communication; FDA, U.S. food and drug administration; FSPTCA, family smoking prevention and tobacco control act; GLB, gay, lesbian, or bisexual; NCTP(s), non-cigarette tobacco product(s); PATH, population assessment of tobacco and health; TCORS, tobacco centers of regulatory science; U.S., United States of America.

## Appendix A

**Table 5:** Constituent panels

Constituent Panel	Constituent 1	Constituent 2	Constituent 3	Constituent 4
1	lead	toluene	1-aminonaphthalene	crotonaldehyde
2	nicotine	hydrogen cyanide	isoprene	acrylonitrile
3	formaldehyde	benzo-a-pyrene	napthalene	NNK
4	arsenic	benzene	acrolein	2-aminonaphthalene
5	carbon monoxide	uranium	1,3-butadiene	N-nitrosonornicotine
6	ammonia	acetaldehyde	nitrosamine	4-aminobiphenyl
