# Erratum to: understanding how perceptions of tobacco constituents and the FDA relate to effective and credible tobacco risk messaging: a national phone survey of U.S. adults, 2014–2015

**DOI:** 10.1186/s12889-017-4760-3

**Published:** 2017-09-20

**Authors:** Marcella H. Boynton, Robert P. Agans, J. Michael Bowling, Noel T. Brewer, Erin L. Sutfin, Adam O. Goldstein, Seth M. Noar, Kurt M. Ribisl

**Affiliations:** 1 Department of Health Behavior, Gillings School of Global Public Health, University of North Carolina at Chapel Hill (UNC), CB #7440, Chapel Hill, NC 27599-7440 USA; 2 Lineberger Comprehensive Cancer Center, UNC, CB #7295, Chapel Hill, NC 27599-7295 USA; 3 Carolina Survey Research Laboratory, UNC, 730 Martin Luther King Jr Blvd, Chapel Hill, NC 27514 USA; 4 Department of Biostatistics, Gillings School of Global Public Health, CB #74203101 McGavran-Greenberg Hall, Chapel Hill, NC 27599-7420 USA; 5 Department of Family Medicine, School of Medicine, UNC, CB #7595Manning Drive, Chapel Hill, NC 27599-7595 USA; 6 Department of Social Sciences and Health Policy, Wake Forest School of Medicine, Medical Center Boulevard, Winston-Salem, NC 27157 USA; 7 School of Media and Journalism, UNC, CB #3365, Chapel Hill, NC 27599-7440 USA

## Erratum

As reported in the original paper [1], the Center for Regulatory Research on Tobacco Communication conducted a telephone survey in 2014–2015 with a national sample of adults ages 18 and older living in the United States (*N* = 5014). Poverty level was determined using the household size and income reported by the respondents and applying the federal poverty numbers available from the U.S. Department of Health and Human Services in 2014. A coding error was made during the data recoding process such that 2.7% of respondents (*n* = 129) were incorrectly classified as living above the poverty line. Below are updated Tables 1, 2 and 4 presenting both the original and corrected estimates. No substantive conclusions reported in the paper were affected by this correction.

**Table 1:** Demographic characteristics as compared to U.S. Census and other national surveys, CRRTC National Adult (≥18 years) Phone Survey 2014–2015

	Unweighted	Weighted		National estimate
	% (n)	%	95% CI	%
ORIGINALLY REPORTED ESTIMATES				
Household Poverty				
At or above federal poverty level	84.0% (3901)	85.7%	(83.8–87.5)	84.6% [2]
Below federal poverty level	16.0% (745)	14.3%	(12.5–16.2)	15.4% [2]
CORRECTED ESTIMATES				
Household Poverty				
At or above federal poverty level	81.3% (3772)	82.5%	(80.4–84.7)	84.6% [2]
Below federal poverty level	18.7% (868)	17.5%	(15.3–19.6)	15.4% [2]

[2] US Census

**Table 2:** Percentage of smokers by selected demographic characteristics, CRRTC National Adult (≥18 years) Phone Survey 2014–2015

	Weighted		National estimate	
	%	95% CI	%	95% CI
ORIGINALLY REPORTED ESTIMATES				
Household Poverty				
At or above federal poverty level	15.4%	(13.5–17.3)	15.2%	(14.6–15.9)
Below federal poverty level	29.3%	(23.9–34.7)	29.2%	(27.5–31.0)
CORRECTED ESTIMATES				
Household Poverty				
At or above federal poverty level	15.1%	(13.2–17.1)	15.2%	(14.6–15.9)
Below federal poverty level	27.9%	(22.9–32.9)	29.2%	(27.5–31.0)

**Table 4:** Subset of Communication-related Variables – CRRTC National Adult Phone Survey 2014–2015

	Weighted Proportion or *M* with 95% Confidence Interval		
	ORIGINALLY REPORTED ESTIMATES	ORIGINALLY REPORTED ESTIMATES	CORRECTED ESTIMATES
	Total	Living in poverty	Living in poverty
Information Seeking			
Have you ever looked for information on chemicals in cigarettes and cigarette smoke?			
Yes	27.5% (25.4–29.7)	25.7% (19.7–31.6)	24.1% (18.8–29.4)
No	72.5% (70.3–74.6)	74.3% (68.4–80.3)	75.9% (70.6–81.2)
In which 1 of these 3 places would you most like to see information on chemicals in cigarettes and cigarette smoke?			
On cigarette packs	54.8% (52.4–57.3)	54.3% (47.4–61.2)	54.6% (48.0–61.2)
In stores	15.0% (13.2–16.7)	18.3% (13.2–23.5)	18.3% (13.6–23.1)
Online	28.7% (26.5–30.9)	25.5% (19.7–31.4)	25.5% (20.2–30.9)
Doesn’t know, refused, or doesn’t want information	1.5% (0.9–2.08)	1.8% (0.3–3.3)	1.5% (0.2–2.7)
Constituent Awareness			
Aware of 0 of 4 constituents in cigarette smoke	37.5% (35.0–40.1)	43.1% (35.7–50.4)	42.5% (35.5–49.5)
Aware of 1 of 4 constituents in cigarette smoke	35.8% (33.4–38.2)	34.7% (28.2–41.2)	33.6% (27.6–39.7)
Aware of 2 of 4 constituents in cigarette smoke	18.7% (16.7–20.7)	13.6% (9.8–17.5)	15.4% (9.3–21.6)
Aware of 3 of 4 constituents in cigarette smoke	5.6% (4.6–6.5)	5.7% (3.0–8.4)	4.9% (2.7–7.2)
Aware of 4 of 4 constituents in cigarette smoke	2.4% (1.7–3.1)	2.9% (0.8–5.1)	3.5% (1.4–5.7)
Knowledge of and Trust for FDA and U.S. Federal Government			
Have you ever heard of the FDA or Food and Drug Administration?			
Yes	94.6% (93.4–95.8)	**87.5% (83.0–92.0)**	**87.1% (83.0–91.2)**
No	5.4% (4.2–6.6)	**12.5% (8.0–17.0)**	**12.9% (8.8–17.0)**
Can the FDA effectively regulate tobacco products?			
Yes	65.2% (62.6–67.8)	67.8% (61.3–74.1)	69.9% (63.9–75.9)
No	34.8% (32.2–37.4)	32.2% (25.8–38.7)	30.1% (24.1–36.1)
How much trust do you have in the federal government? *M score, 0 = none at all - 4 = a great deal*	2.0 (1.9–2.0)	**2.2 (2.0–2.4)**	**3.1 (2.9–3.2)**

Note. Point estimates in **bold** text were found to be significantly different from their respective comparison group (e.g., smokers were compared to non-smokers, young adults compared to older adults, etc.) using either PROC SURVEYFREQ or PROC SURVEYREG to make the comparisons
